# Metabolic remodeling and immune evasion in glioblastoma: a focus on serine and lipid networks

**DOI:** 10.3389/fonc.2026.1676560

**Published:** 2026-02-03

**Authors:** Dongxin Jiang, Chuheng Wang, Yao Zhao, Yunqian Li

**Affiliations:** Department of Neurosurgery, The First Hospital of Jilin University, Changchun, China

**Keywords:** fatty acid metabolism, glioblastoma, liver X receptors, metabolic intervention, phosphoglycerate dehydrogenase, serine, tumor microenvironment

## Abstract

Glioblastoma (GBM) is a highly aggressive brain tumor characterized by metabolic plasticity that fuels growth, therapy resistance, and immune evasion. Among its reprogrammed pathways, serine and lipid metabolism play central roles. The serine synthesis pathway (SSP)—via PHGDH, PSAT1, and SHMT2—supports nucleotide biosynthesis, redox balance, and epigenetic regulation, especially under hypoxic and nutrient-deprived conditions. Meanwhile, fatty acid flux, FABP7-mediated PUFA transport, and cholesterol uptake reshape the tumor microenvironment, sustain glioma stemness, and promote immune suppression. Key lipid enzymes and ferroptosis regulators such as MAGL, ACSL4, and xCT modulate tumor survival and therapy response. GBM cells also exhibit high reliance on exogenous cholesterol, with dysregulation of LXR–SREBP pathways and mevalonate flux contributing to autophagy and proliferation. Therapeutic strategies targeting metabolic vulnerabilities—including SSP blockade, cholesterol homeostasis disruption, and ferroptosis induction—show synergistic effects with conventional agents like temozolomide. This review highlights the intertwined metabolic circuits in GBM and explores their translational potential as targets for precision therapy.

## 1 Introduction

Glioblastoma (GBM) is the most aggressive and lethal primary malignancy of the central nervous system, characterized by diffuse infiltration, rapid proliferation, and pronounced therapeutic resistance (1, 2). While traditional studies have focused on oncogenes and signaling pathways, recent evidence positions metabolic reprogramming as a key driver of glioma pathogenesis, enabling adaptation to hypoxia, nutrient scarcity, and immunologic stress (3, 4). Among the diverse metabolic networks altered in GBM, serine and lipid metabolism emerge as closely intertwined hubs that collectively support tumor progression (5–8). The serine synthesis pathway (SSP)—via enzymes such as phosphoglycerate dehydrogenase (PHGDH), phosphoserine aminotransferase 1 (PSAT1), and serine hydroxy methyltransferase (SHMT2)—fuels nucleotide synthesis, NADPH generation, and one-carbon metabolism (9–11). In parallel, lipid metabolic rewiring, encompassing fatty acid elongation, desaturation, and cholesterol trafficking, provides the structural and energetic scaffolding for membrane formation, signal transduction, and immune modulation (12).

Importantly, serine and lipid metabolism are not independent circuits; rather, they converge at multiple regulatory and functional levels (13). For example, SSP-derived NADPH and S-adenosylmethionine (SAM) are indispensable for fatty acid desaturation and phospholipid methylation, while acetyl-CoA and folate intermediates link glycolysis, serine, and lipogenic flux (14). These integrated pathways are co-regulated by stress-adaptive transcription factors, including ATF4 and HIF-1α, and cooperate to maintain glioma stemness, redox homeostasis, and resistance to ferroptosis and chemotherapy (15, 16). Given the rising therapeutic interest in both SSP inhibitors (PHGDH, SHMT2) and lipid metabolism modulators (SCD1, FASN, LXR agonists), a comprehensive understanding of their metabolic and immunological crosstalk is urgently needed (6, 9, 10, 17). This review aims to synthesize current insights into serine–lipid metabolic integration in GBM, identify key regulatory nodes, and explore translational implications for precision therapy.

## 2 Roles of fatty acid metabolism in glioblastoma

### 2.1 Fatty acid metabolism remodels the tumor microenvironment

Fatty acids serve dual roles as substrates for β−oxidation and as structural constituents of phospholipids, glycolipids and cholesterol (18, 19). Critically, they also act as precursors for lipid mediators including eicosanoids and lysophosphatidic acid; dysregulated production of these molecules activates inflammatory signaling and drives GBM progression via RAS/PI3K/RHO pathways (20, 21). The GBM microenvironment, composed of neoplastic, stromal and immune cells such as tumour−associated macrophages (TAMs), is metabolically remodeled by fatty acid fluxes (22). Therapy resistance in GBM arises largely from glioblastoma stem cells (GSCs) (23, 24). TAMs promote tumour evolution through the ARS2–MAGL axis, wherein MAGL overexpression releases free fatty acids from monoacylglycerols, thereby fueling GSC self−renewal and prostaglandin E_2_ (PGE_2_) synthesis (25). PGE_2_ polarizes TAMs toward an immunosuppressive phenotype, characterized by impaired antigen presentation, increased secretion of IL−10 and TGF−β, and extracellular matrix remodelling (26, 27). These immunosuppressive TAMs in turn reinforce GSC stemness, invasion and neovascularization (28–30). By contrast, immunostimulatory TAMs—which exhibit elevated pro−inflammatory cytokine production, antigen−presenting capacity and cytotoxic activity—are linked to anti−tumour immunity (31). PGE_2_ further amplifies GSC self−renewal via pLRP6/β−catenin (Wnt) signaling, an effect reversible by the selective MAGL inhibitor JZL184 (25). Hypoxic niches, a hallmark of GBM, enhance Ras−dependent uptake of exogenous fatty acids. Under these conditions, stearoyl−CoA desaturase 1 (SCD1) sustains the oleic/stearic acid balance essential for membrane fluidity and cellular integrity (32). Suppression of glioma−enriched Ras−GRF1 induces apoptosis through the H−Ras/ERK cascade, highlighting its therapeutic potential (33, 34).

### 2.2 Fatty acid–binding proteins and other regulators in GBM

**Figure 1:**
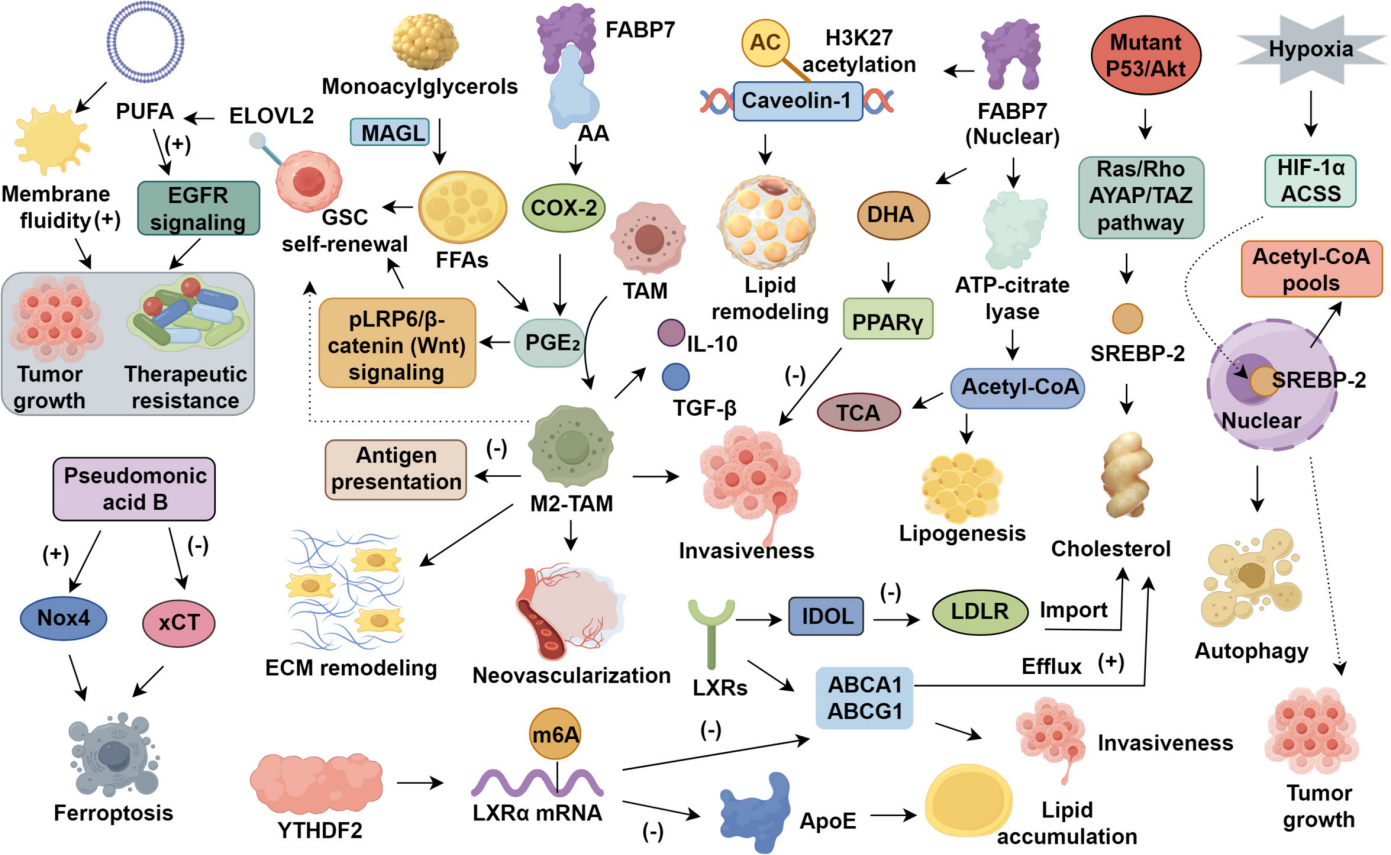
Serine and lipid networks in glioblastoma.

Fatty acid–binding proteins (FABPs) coordinate the intracellular trafficking of polyunsaturated fatty acids (PUFAs). Among these, FABP7 is highly expressed in astrocytes and neural progenitors (35, 36) and is upregulated in gliomas, where it exerts compartment-specific effects on tumor progression (37, 38). Cytoplasmic FABP7 complexes with arachidonic acid (AA) to enhance COX−2−dependent prostaglandin production, promoting tumor motility and metastasis. In contrast, nuclear FABP7 binds docosahexaenoic acid (DHA) and activates PPARγ signaling, which restrains invasion (39). Nuclear FABP7 also upregulates ATP−citrate lyase, supplying acetyl−CoA for the TCA cycle and *de novo* lipogenesis to sustain glioma bioenergetics. Epigenetically, FABP7 drives histone H3K27 acetylation at the caveolin−1 promoter, enhancing its transcription and facilitating lipid remodeling during gliomagenesis (40). In glioma stem cells, super−enhancer−driven expression of ELOVL2 enriches PUFA−containing phospholipids, increasing membrane fluidity and amplifying EGFR signaling to promote growth and therapy resistance (41). Ferroptosis, an iron−dependent cell death triggered by lipid peroxidation, represents a promising therapeutic avenue in GBM (42). Pseudomonic acid B induces ferroptosis by upregulating Nox4 and inhibiting the cystine/glutamate antiporter SLC7A11 (xCT). xCT knockdown similarly sensitizes cells to ferroptosis and synergizes with temozolomide (43, 44). The enzyme ACSL4 facilitates incorporation of PUFAs into phospholipids, thereby generating substrates for lipid peroxidation (45). ACSL4 overexpression further suppresses GPx4 activity, amplifying oxidative stress and ferroptotic cell death (46) (**Figure 1**).

### 2.3 Cholesterol metabolism in GBM

Cholesterol, a critical determinant of membrane integrity, is synthesized *de novo* through the mevalonate pathway, with 3-hydroxy-3-methylglutaryl-coenzyme A (HMG-CoA) reductase functioning as the key regulatory enzyme. Acetyl-CoA serves as the initial substrate, leading sequentially to HMG-CoA, mevalonate, and farnesyl pyrophosphate—a central precursor for cholesterol as well as for ubiquinone, heme A, and protein prenylation (47, 48). Within the central nervous system (CNS), which harbors approximately 20–25% of total body cholesterol, neural cells rely predominantly on local synthesis due to the impermeability of the blood–brain barrier to circulating lipoproteins; astrocytes are the principal producers (49). Newly synthesized cholesterol is packaged into ApoE-containing low-density lipoprotein (LDL)-like particles, internalized via LDL receptors (LDLR), and hydrolyzed within lysosomes. Additionally, astrocytes export cholesterol to neurons through ApoE-rich HDL-like particles in a process mediated by ABCA1 and ABCG1 transporters (50).

## 3 Cholesterol homeostasis and lipid raft signaling in GBM

### 3.1 Sterol regulatory element-binding proteins and liver X receptors

Cholesterol homeostasis in GBM is primarily regulated through the counterbalanced activities of SREBPs and liver X receptors (LXRs) (51). SREBP-2, in particular, directs cholesterol synthesis and uptake, whereas SREBP-1 isoforms modulate fatty acid metabolism (52). Oncogenic pathways involving mutant p53 and Akt enhance SREBP-2 activity, thereby activating Ras, RhoA (53), and YAP/TAZ signaling, which amplifies cholesterol production (54). The hypoxic and acidotic tumour microenvironment further stimulates nuclear translocation of SREBP-2 via HIF-1α and ACSS2, preserving acetyl-CoA levels to support autophagy and tumor growth (52, 55). Low-grade gliomas display elevated expression of cholesterol-related genes, implying that metabolic reprogramming may precede malignant transformation (56). LXRs counterbalance cholesterol accumulation by promoting efflux through ABCA1/ABCG1 and limiting LDLR-mediated import via induction of the E3 ligase IDOL (57). LXRα/β form obligate heterodimers with retinoid X receptors (RXRs) to regulate glia–neuron cholesterol transport, with neuronal compartments exhibiting enhanced efflux. GBM cells, unlike normal astrocytes, exhibit elevated LDLR expression, increased lipid uptake, and reduced endogenous LXR agonist levels, rendering them particularly sensitive to LXR activation (58). Additionally, epitranscriptomic control via m6A methylation reprograms cholesterol metabolism: YTHDF2-mediated degradation of LXRα mRNA represses ABCA1, ABCG1, and ApoE expression, promoting lipid accumulation and tumor progression (59, 60).

### 3.2 3-hydroxy-3-methylglutaryl-coenzyme A reductase (HMGCR) and lipid rafts

Upregulation of HMGCR drives GBM proliferation and invasiveness through activation of the Hippo pathway effector TAZ and subsequent induction of CTGF. Conversely, pharmacologic or genetic inhibition of HMGCR limits tumor dissemination *in vivo (*60, 61). Statins, as HMGCR inhibitors, attenuate these malignant phenotypes, underscoring HMGCR as a potential therapeutic target (17). However, their efficacy in GBM remains limited, as tumor cells primarily depend on exogenous cholesterol uptake, unlike astrocytes, thereby reducing sensitivity to enzymatic blockade (62). The interaction between statins and temozolomide (TMZ) remains controversial, with reports indicating either reduced cytotoxicity or enhanced apoptotic activity (63, 64). These discrepancies appear to arise from disruptions in mevalonate pathway–regulated autophagy, rather than cholesterol depletion. Rab11 (a small GTPase) geranylgeranylation is essential for autophagosome formation; thus, inhibition of mevalonate flux impairs Rab11-mediated autophagic turnover and autophagosome–lysosome fusion, ultimately sensitizing GBM cells to TMZ-induced apoptosis (65). Lipid rafts serve as organizing centers for oncogenic signaling and apoptotic cascades (66, 67). In GBM, lipid rafts are critical for compartmentalization of death receptor 5 (DR5), which recruits FADD and caspase−8 to initiate extrinsic apoptosis and contributes to TMZ cytotoxicity (63, 68, 69). Cholesterol scaffolding within rafts facilitates DR5 clustering and DISC assembly. Elevated cholesterol enhances DR5 activation, and TMZ upregulates DR5 expression, suggesting that combining TMZ with strategies to enrich membrane cholesterol may potentiate raft-dependent apoptotic signaling (70).

## 4 SSP metabolism and key enzymes in glioma progression

### 4.1 Functions of serine

Cellular serine originates from extracellular uptake, glycine interconversion, or *de novo* synthesis via the serine synthesis pathway (SSP), a frequently dysregulated route in malignancies that promotes proliferation, therapy resistance, and poor prognosis (71). Transcriptional control of the SSP is coordinated by activating transcription factor 4 (ATF4), which upregulates key enzymes including PHGDH, PSAT1, PSPH, and SHMT (72, 73). Under serine limitation, ATF3 enhances this program by binding enhancer or promoter regions of SSP genes, amplifying adaptive responses (74). Transcription factor CP2 (TFCP2) further acts as an essential co-regulator; its depletion suppresses PHGDH expression and impairs glioma proliferation and tumorigenicity (75). Serine metabolism sustains tumors through multiple mechanisms: (i) supplying biosynthetic precursors, (ii) fueling mitochondrial folate-mediated one−carbon cycling to generate S−adenosylmethionine (SAM) for epigenetic methylation, and (iii) maintaining NADPH production via one−carbon metabolism to support redox balance and nucleotide synthesis (76). One−carbon units (methylene, methyl, and formyl groups) are shuttled by tetrahydrofolate (THF), linking serine catabolism to nucleotide biosynthesis and glutathione-dependent antioxidant systems (77). Serine serves as the major one−carbon donor: its conversion to glycine releases a methylene group into the folate cycle, driving SAM production and subsequent DNA/histone methylation (78, 79). In glioblastoma’s hypoxic, nutrient−poor niche, elevated serine and glycine levels are observed. Glutamine deprivation further upregulates PSAT1 and SHMT2, diverting serine flux into one−carbon metabolism as a stress−adaptive survival strategy (80). This metabolic flexibility underscores serine’s essential role in maintaining glioma viability under duress.

### 4.2 Key enzymes of the SSP and their association with GBM progression

#### 4.2.1 PHGDH

PHGDH, a key rate-limiting enzyme in the serine synthesis pathway (SSP), is consistently upregulated in multiple malignancies including breast cancer, melanoma, and non−small cell lung cancer (81, 82). In glioma, PHGDH expression correlates with tumor grade, and its inhibition suppresses the transcription of proliferative and invasive mediators such as MMP−2, cyclin D1, VEGF, CHK2, and FOXM1, thereby limiting tumor growth and invasion *in vitro* and *in vivo* (83). In glioma cells with moderate-to−high endogenous PHGDH, silencing reduces the NADPH/NADP^+^ ratio, curbs proliferation, and increases susceptibility to hypoxia−induced apoptosis, whereas forced overexpression enhances hypoxia tolerance and tumorigenicity (84). Elevated PHGDH also contributes to therapy resistance and poor prognosis (85). For instance, PHGDH promotes mTORC1 activation and modulates rapamycin sensitivity during breast cancer lung metastasis (86). Multi−omics analyses reveal that ATF4−mediated transcriptional activation of PHGDH in glioblastoma endothelial cells redirects glycolytic flux toward nucleotide synthesis, fueling endothelial hyperproliferation. PHGDH inhibition alleviates tumor hypoxia, promotes cytotoxic T−lymphocyte infiltration, and augments CAR−T cell efficacy (87). Furthermore, PHGDH stabilizes FOXM1 via direct interaction, and its depletion prolongs survival in glioma xenografts, highlighting the PHGDH–FOXM1 axis as a promising therapeutic target (83).

#### 4.2.2 PSAT1 and PSPH

PSAT1 and PSPH, which catalyze the sequential second and third reactions of the serine synthesis pathway (SSP), are frequently overexpressed in malignancies and promote oncogenic progression (88, 89). In triple−negative breast cancer, high PSAT1 expression correlates with enhanced aggressiveness, metastasis, and poor survival (90). Similarly, PSAT1 is upregulated in epithelial ovarian carcinoma, where its knockdown suppresses clonogenicity, induces G1 arrest, and promotes apoptosis (91). In gliomas, both enzymes are consistently elevated across histological grades compared to non−neoplastic brain tissue. Multiple Gene Expression Omnibus (GEO) datasets, qPCR, and immunoblot analyses confirm their overexpression in glioblastoma cell lines and glioma stem−like cells relative to normal astrocytes (6). Furthermore, in primary glioblastoma, PSAT1 and PSPH levels positively associate with phosphorylated AMPK (pT172) and HIF−1α activity (92). Patients with low PSAT1 or PSPH expression had a median overall survival of 20 weeks, whereas high expression predicted significantly shorter survival. These data underscore the AMPK–HIF−1α–SSP axis as a key driver of glioblastoma pathogenesis and a prognostic indicator (93).

#### 4.2.3 SHMT

Serine hydroxymethyltransferase (SHMT) comprises cytosolic (SHMT1) and mitochondrial (SHMT2) isoforms, each catalyzing the reversible interconversion of serine and tetrahydrofolate (THF) to glycine and 5,10−methylenetetrahydrofolate in distinct subcellular compartments. While SHMT1 participates in cytosolic folate metabolism and pathway regulation (94), SHMT2 predominantly drives mitochondrial one−carbon flux through oxidative serine catabolism, a process tightly coupled to folate transport (95, 96). SHMT2 is frequently overexpressed in malignancies, including hepatocellular and lung carcinomas (97, 98), and elevated SHMT2 expression correlates with poor prognosis in gliomas, serving as an independent prognostic biomarker (99). In glioblastoma, SHMT2 and glycine decarboxylase (GLDC) are enriched within pseudopalisading cells surrounding necrotic regions, promoting survival under hypoxic conditions. SHMT2 further suppresses pyruvate kinase M2 (PKM2) activity and reduces oxygen consumption, enhancing persistence in ischemic niches. When GLDC activity is insufficient to catabolize accumulated glycine, toxic metabolites such as aminoacetone and methylglyoxal are produced, rendering SHMT2−overexpressing cells susceptible to GLDC inhibition (100). These observations establish SHMT2 as a central metabolic node facilitating glioma adaptation to nutrient and oxygen deprivation, highlighting its therapeutic relevance for targeting metabolic plasticity in the tumor microenvironment.

## 5 Emerging therapeutic strategies in GBM

Despite aggressive multimodal therapy—including surgical resection, radiotherapy, and temozolomide—GBM remains clinically intractable, necessitating precision-targeted strategies. Bevacizumab (BEV) is an anti-VEGF antibody for recurrent GBM to reduce angiogenesis and edema (101). Amplification or mutation of EGFR drives tumor proliferation and underlies poor prognosis, yet EGFR-targeted therapies have yielded limited efficacy due to intratumoral heterogeneity and adaptive resistance (102). Regorafenib, a multikinase inhibitor targeting VEGFR, PDGFR, and FGFR, has demonstrated clinical benefit in recurrent GBM and is under clinical evaluation (103). Additional actionable alterations include PTPRZ1–MET fusions responsive to capmatinib and BRAF V600E mutations sensitive to vemurafenib, particularly in pleomorphic xanthoastrocytoma (104). Nevertheless, EGFR-targeted therapies have yielded limited clinical success in GBM, largely due to intratumoral heterogeneity and adaptive resistance (105). Recent insights implicate lipid metabolic rewiring as a crucial modulator of receptor tyrosine kinase activity and drug response (106). ELOVL2-mediated enrichment of long-chain polyunsaturated phospholipids enhances membrane fluidity, promoting EGFR clustering and activation, thereby attenuating TKI efficacy (41, 107). In parallel, FABP7-driven lipid trafficking and COX-2–dependent prostaglandin synthesis amplify the oncogenic cascades, revealing a metabolic–signaling axis that may be therapeutically actionable. These findings support combined inhibition of EGFR and lipid metabolism to overcome resistance and enhance therapeutic durability (17, 108).

GBM exhibits marked metabolic plasticity, extending to serine metabolism. While many tumors rely on exogenous serine (109, 110), GBM adapts to deprivation by activating the serine synthesis pathway (SSP). Serine withdrawal suppresses pyruvate kinase M2 (PKM2), diverting glycolytic intermediates into SSP via ATF4- and G9A-mediated induction of PHGDH, PSAT1 and PSPH (111, 112). As the rate-limiting SSP enzyme, PHGDH is crucial for redox homeostasis and nucleotide synthesis. Concurrent PHGDH targeting and dietary serine/glycine restriction severely impair nucleotide and glutathione production, suppressing tumor growth (113, 114). Notably, neither glycine nor one-carbon donors alone restore proliferation, though combined supplementation partially rescues ATP and GTP levels. SSP dependence varies across GBM lines: LN-308 and LN-229 (PHGDH^−low^) are serine−dependent, whereas G55 (PHGDH^−high^) shows resistance. PHGDH inhibition by CBR-5884 under –SG conditions further suppress proliferation and exacerbates hypoxia−induced cytotoxicity (115). The selective PHGDH inhibitor NCT503 synergizes with temozolomide to induce apoptosis in chemoresistant, MGMT−expressing models (116). Regorafenib stabilizes PSAT1 and concurrently inhibits autophagic flux through PRKAA activation and RAB11A disruption; PSAT1 levels correlate with drug sensitivity (117). Beyond serine metabolism, targeting lipid biosynthetic enzymes represents an emerging therapeutic axis. Fatty acid synthase (FASN)—upregulated in high−grade gliomas—drives palmitate synthesis for membrane biogenesis and oncogenic signaling (118, 119); its inhibitor TVB-2640 shows preclinical efficacy. SCD1, which modulates fatty acid saturation and membrane fluidity, also supports glioma survival (32, 120). SCD1 inhibition increases oxidative susceptibility and may synergize with ferroptosis inducers or chemotherapy (121, 122). Collectively, FASN and SCD1 represent promising targets for combinatorial approaches aimed at disrupting GBM’s lipid−mediated adaptive capacity.

## 6 Conclusion

Glioblastoma exhibits profound metabolic plasticity, in which serine and lipid metabolic rewiring collectively sustains tumor proliferation, redox homeostasis, immune evasion, and resistance to therapy. The serine synthesis pathway, through PHGDH, PSAT1, and SHMT2, supports nucleotide and glutathione production while fueling one-carbon metabolism, SAM-dependent methylation, and NADPH generation. In parallel, aberrant lipid metabolism, including fatty acid elongation, desaturation, and cholesterol accumulation, drives glioma stemness, reshapes the immunosuppressive microenvironment, and modulates ferroptosis sensitivity. Importantly, serine-derived metabolites such as NADPH and SAM act as metabolic bridges linking amino acid and lipid biosynthesis, establishing a tightly coordinated metabolic–epigenetic network that enables glioma cells to adapt to hypoxia and nutrient deprivation.

This review underscores the clinical potential of targeting serine–lipid metabolic crosstalk in GBM. Inhibitors of SSP enzymes (PHGDH, SHMT2), fatty acid metabolism (FASN, SCD1), and cholesterol homeostasis (LXR agonists) have shown synergistic effects when combined with chemotherapy, ferroptosis inducers, or autophagy blockers. As spatial and single-cell multi-omics technologies evolve, uncovering metabolic heterogeneity across tumor subtypes and immune niches will be essential for refining metabolic vulnerabilities. By dissecting the convergent metabolic dependencies that sustain glioma aggressiveness, future therapeutic strategies may exploit these rewired circuits to overcome resistance and achieve durable clinical responses in GBM.
